# Beyond Standard Algorithms: Diagnostic, Procedural, and Analgesic Challenges in Refractory Postpartum Hemorrhage

**DOI:** 10.7759/cureus.101973

**Published:** 2026-01-21

**Authors:** Fiona Lin, Hibah Mohammed, Adib Khan, Julia R Legiec, Hussain Rawiji

**Affiliations:** 1 Medicine, Lake Erie College of Osteopathic Medicine, Bradenton, USA; 2 Medicine, Lake Erie College of Osteopathic Medicine, Jacksonville, USA; 3 Obstetrics and Gynecology, Lake Erie College of Osteopathic Medicine, Bradenton, USA; 4 Pediatrics and Obstetrics, AdventHealth Central Florida, Orange City, USA

**Keywords:** neuraxial anesthesia, obstetric anesthesia, postpartum hemorrhage, procedural analgesia, uterine atony, uterine contractility, uterine vacuum device

## Abstract

Postpartum hemorrhage (PPH) remains a leading cause of preventable maternal morbidity and mortality, with management often complicated by overlapping etiologies, delayed manifestations of hypovolemia, and procedural challenges related to analgesia availability. We report a case of recurrent PPH in a 24-year-old gravida 2, para 2 woman following spontaneous vaginal delivery without neuraxial anesthesia, in whom persistent bleeding refractory to uterotonic therapy was associated with discordant findings of a firm uterine fundus and a boggy lower uterine segment. Hemorrhage control was delayed by significant procedural pain during placement of an intrauterine vacuum-induced hemorrhage-control device, leading to continued bleeding and hemodynamic compromise with an estimated blood loss of 1,400-1,500 mL and activation of a massive transfusion protocol. Hemostasis was achieved with repeat device placement and blood product transfusion, followed by clinical stabilization. This case highlights the importance of recognizing localized uterine atony, the limitations of visual blood loss assessment, and the critical role of neuraxial anesthesia and analgesia planning in facilitating timely intrauterine interventions during acute obstetric hemorrhage.

## Introduction

Postpartum hemorrhage (PPH) accounts for about 25% of maternal deaths worldwide and remains a leading cause of preventable maternal mortality [1]. PPH is defined as cumulative blood loss of 1,000 mL or more or blood loss accompanied by signs or symptoms of hypovolemia within 24 hours after delivery, regardless of route [1,2]. Accurate diagnosis remains challenging because visual estimation demonstrates only limited sensitivity for detecting PPH ≥500 mL, and clinical signs of hypovolemia often do not manifest until blood loss exceeds 1,500 mL [1,2].

The causes of PPH include uterine atony (70% of cases), trauma (lacerations or rupture, 10-20%), tissue (retained placenta or clots, 10-15%), and thrombin (coagulopathy, 5%) [1-3]. A critical diagnostic nuance is recognizing that many concurrent causes frequently contribute to PPH [4]. The finding of a boggy lower uterine segment, despite a firm fundus, represents a particularly challenging scenario, suggesting localized atony or retained tissue despite apparent overall contractility, which requires manual intrauterine exploration and clot evacuation [2].

Intrauterine vacuum-induced hemorrhage control devices represent a significant advancement in managing refractory PPH. The Jada System employs low-level vacuum, usually at a negative pressure of -80 mmHg, to mechanically induce uterine myometrial contraction and collapse the uterine walls, reducing blood flow to the placental bed [5,6]. Unlike balloon tamponade devices, which apply outward pressure, vacuum suction reduces uterine volume, promoting the coiling of spiral arteries and restoration of tone [7]. The device has a high treatment success rate with a hemorrhage control time as rapid as three minutes and has been specifically successful in isolated atony cases [5,8].

Despite demonstrated efficacy, anesthetic and procedural pain management during intrauterine device placement in acute PPH settings remains inadequately addressed in the literature. The use of IV opioids such as fentanyl for procedural analgesia represents a pragmatic approach when regional anesthesia is not in place, even though evidence-based dosing protocols specific to this indication are lacking [5]. The American Society of Anesthesiologists recommends titrating medications incrementally to the lowest required doses of opioids to produce the desired degree of anxiolysis, with minimal sedation being ideal [8]. More anesthesia-based protocols are necessary for PPH when pain management becomes a significant issue for using the Jada device.

Overall, this case report illustrates the critical diagnostic subtleties, including discordant lower uterine segment atony with firm fundus and procedural challenges of device placement without adequate anesthesia.

## Case presentation

In September 2024, a 24-year-old gravida 1, para 1 (G1P1001) female, with one prior term pregnancy resulting in one living child, delivered at 38 weeks and two days of gestation. She had a spontaneous vaginal delivery of a viable female infant with APGAR scores of 9 at one minute and 9 at five minutes. Labor progressed to full cervical dilation after approximately 10 hours and 15 minutes of active labor. The patient delivered in the dorsal lithotomy position, and a vacuum-assisted vaginal delivery was performed under epidural anesthesia. PPH was suspected, and management was initiated. Manual exploration was performed, and blood clots were removed from the uterus. The Jada device was placed without complications. Quantitative blood loss after resolution of bleeding was estimated at 1,255 mL.

Ten months later, the now G2P2002 patient delivered her second child at 9:05 AM: a viable male infant at 38 weeks and six days of gestation. APGAR scores were 8 at one minute and 9 at five minutes, following 45 minutes of pushing without epidural anesthesia. After this delivery, the patient continued to bleed and was initially treated with carboprost (Hemabate 250 mcg/mL IM), misoprostol (Cytotec 1000 mcg rectally), and tranexamic acid (1 g IV). At this time, her systolic blood pressure was consistently in the 140s for about three minutes since the time of delivery (Table 1). Her blood pressure increased to 170/127 with a heart rate of 144 beats/min another three minutes later (Table 1). After medical management, the patient’s blood loss was significantly reduced to slow but persistent bleeding. Methylergonovine (Methergine 0.2 mg IM) was administered, and the estimated blood loss was measured at 700 mL.

**Table 1:** Heart rate and blood pressure during acute PPH in the immediate post-delivery period PPH, postpartum hemorrhage

Time	9:05 am	9:06 am	9:07 am	9:10 am	9:14 am	9:15 am	9:16 am	9:19 am
Heart rate (beats/min)	132	130	130	110	144	136	121	124
Blood pressure (mmHg)	-	111/52	142/69	170/127	121/101	-	134/76	131/80
Time	9:20 am	9:22 am	9:25 am	9:26 am	9:29 am	9:30 am	9:35 am	9:40 am
Heart rate (beats/min)	122	115	108	-	113	111	115	124
Blood pressure (mmHg)	-	130/82	128/74	-	117/92	-	129/75	-

A bimanual examination was performed, which revealed a boggy lower uterine segment despite a firm uterine fundus on external abdominal palpation. Approximately 200-300 mL of clots were manually evacuated while the patient was awake, yet uncooperative. She did not present with any signs of altered mental status during the PPH. One unit of blood was ordered for immediate transfusion. Continuous bleeding prompted the decision to place a Jada uterine vacuum device. Placement of the Jada was challenging due to patient discomfort and positioning difficulties. She experienced significant pain during insertion and repeatedly elevated her pelvis off the bed when the device was attempted to be placed. This made it difficult to maintain a dorsal lithotomy position. After explaining the serious nature of this life-threatening situation, the patient was able to maintain the dorsal lithotomy position, and the Jada device was successfully placed. Fentanyl (100 mcg IV) was then administered due to expressed pain as shown through continued patient resistance.

Despite medical management, clots subsequently recurred. A speculum examination was performed, which demonstrated no evidence of active bleeding or hematoma within the upper third of the vagina or at the cervical os. Clots were evacuated, and another Jada uterine vacuum device was placed. At this time point, the estimated blood loss was approximately 1,400-1,500 mL, and a massive transfusion protocol was initiated. The patient initially became symptomatic with dizziness, at which time the first unit of blood was administered. Additionally, she received approximately 3 L of IV crystalloid fluid boluses. The Jada device remained in place and functioned effectively for 24 hours until it was removed the next morning. In total, per hospital protocols, the patient received two units of packed red blood cells and one unit of fresh frozen plasma, with a favorable clinical response and stabilization of blood pressures that were trending systolic pressures in the 120s and 110s at around 9:29 am to 9:35 am (Table 1). Postpartum iron therapy was initiated, including iron sucrose administration, for the management of post-hemorrhagic anemia (Table 2).

**Table 2:** Follow-up CBC 23 hours after PPH PPH, postpartum hemorrhage

Parameter (units)	Lab value	Reference range
White blood cells (K/µL)	12.28	4.8-10.8
Red blood cells (M/µL)	2.88	4.2-5.4
Hemoglobin (g/dL)	8.1	12.0-16.0
Hematocrit (%)	25.3	37.0-47.0
Mean corpuscular volume (fL)	87.8	80.0-99.0
Mean corpuscular hemoglobin (pg)	28.1	27.0-33.0
Mean corpuscular hemoglobin concentration (g/dL)	32	33.0-36.5
Red cell distribution width (%)	19.2	11.9-17.7
Platelet count (K/µL)	121	150-400
Mean platelet volume (fL)	10.9	7.4-10.4
Nucleated red blood cells %	0	0.0-0.0

## Discussion

This case highlights critical diagnostic and anesthetic challenges in recurrent PPH management, particularly the importance of neuraxial anesthesia for facilitating emergent interventions, appropriate hemodynamic monitoring strategies, and the diagnostic significance of discordant examination findings.

The bimanual examination finding of a boggy lower uterine segment despite a firm uterine fundus represents localized atony of the lower uterine segment despite apparent overall contractility [4]. ACOG guidelines specify that when the fundus is firm, but the lower segment is dilated and atonic, the approach is manual clot removal and bimanual compression while awaiting uterotonic response, with intrauterine tamponade considered if persistent [4]. This underscores that multiple concurrent causes frequently contribute to PPH, necessitating systematic evaluation even when one etiology appears evident [4,5]. In this case, in which there could be many causes that could contribute to PPH, hemodynamic monitoring and pain management remain crucial for diagnosis and treatment.

Hemodynamic monitoring in PPH should be individualized based on hemorrhage severity and risk [6]. Standard monitoring includes continuous pulse oximetry, cardiac rhythm monitoring, frequent vital sign assessment, urine output measurement, and laboratory evaluation of coagulation and metabolic parameters [6-8]. Invasive arterial blood pressure monitoring is indicated in cases of significant blood loss or hemodynamic instability to allow continuous, beat-to-beat assessment and guide resuscitation [4]. For patients at high risk of severe PPH, early arterial and central venous access should be considered, with radial arterial cannulation preferred due to increased thrombotic risk at femoral sites in pregnant and postpartum patients [4,6]. In this case, as blood loss approached 1,400-1,500 mL with tachycardia and hypertension, arterial line placement would have enabled real-time blood pressure monitoring and earlier detection of cardiovascular decompensation [9].

Initial PPH management includes supplemental oxygen and continuous pulse oximetry to maintain tissue oxygenation and detect hypoxemia related to hypovolemia or anemia [6,8,10]. Postpartum patients remain at increased risk of aspiration due to delayed gastric emptying and elevated intra-abdominal pressure [8]. When general anesthesia is required, airway management is challenging because pregnancy-related physiologic changes increase the risk of failed intubation and rapid desaturation; standard practice includes preoxygenation, rapid-sequence induction, and ready access to videolaryngoscopy [4].

In contrast to septic shock, vasopressors play a limited role in hemorrhagic shock, where hemorrhage control and volume replacement are the primary interventions [11]. Vasopressors should be reserved for refractory hypotension despite adequate resuscitation, as premature use may obscure ongoing bleeding and worsen tissue hypoperfusion [11,12]. When indicated, norepinephrine is preferred with individualized perfusion targets. In this case, hypertension and tachycardia reflected compensatory sympathetic responses rather than an indication for vasopressor therapy, and management appropriately focused on volume control.

In this case, the lack of neuraxial anesthesia significantly complicated the management of PPH. Labor neuraxial analgesia is associated with reduced severe maternal morbidity and PPH risk, largely because an epidural catheter allows rapid conversion to procedural anesthesia for invasive uterine interventions, facilitating timely hemorrhage control [13]. In this case, severe pain during intrauterine device placement led to patient movement and delayed definitive management, requiring IV opioid administration after the procedure; with an epidural in place, effective anesthesia could have been achieved rapidly [14]. American Society of Anesthesiologists guidelines recommend neuraxial anesthesia only in hemodynamically stable patients, with general anesthesia reserved for major hemorrhage with instability [15]. Although regional anesthesia is preferred for PPH interventions, general anesthesia is more commonly used in severe cases and may increase blood loss due to the uterine-relaxant effects of volatile anesthetics [12,16]. Given this patient’s elevated risk for recurrent PPH, proactive neuraxial analgesia should be considered in future pregnancies, and when unavailable, carefully titrated procedural analgesia should be administered before device placement to minimize respiratory complications [3,4,15].

## Conclusions

This case highlights the importance of recognizing diagnostic subtleties in PPH, including localized lower uterine segment atony despite a firm fundus, and the procedural challenges encountered when neuraxial anesthesia is unavailable. Although intrauterine vacuum-induced hemorrhage-control devices are effective, inadequate analgesia may delay deployment and compromise hemorrhage control. Patients with prior PPH represent a high-risk group in whom anticipatory neuraxial analgesia and early anesthetic involvement should be considered. Incorporating structured analgesia planning, comprehensive uterine assessment, and individualized hemodynamic monitoring into PPH protocols may improve procedural efficiency, patient tolerance, and clinical outcomes.
